# Smoking-related cue reactivity in a virtual reality setting: association between craving and EEG measures

**DOI:** 10.1007/s00213-020-05733-3

**Published:** 2020-12-02

**Authors:** Stefano Tamburin, Denise Dal Lago, Federica Armani, Marco Turatti, Riccardo Saccà, Simone Campagnari, Cristiano Chiamulera

**Affiliations:** 1 Department of Neurosciences, Biomedicine and Movement Sciences, University of Verona, Piazzale Scuro 10, I-37134 Verona, Italy; 2 Department of Diagnostic and Public Health, University of Verona, Piazzale Scuro 10, I-37134 Verona, Italy

**Keywords:** Craving, Cue reactivity, Electroencephalography, Smoking, Virtual reality, Visual analogue scale (VAS)

## Abstract

**Background:**

Cue-reactivity is the array of responses that smokers exhibit when exposed to conditioned and contextual stimuli previously associated to substance use. The difficulty to experimentally recreate the complexity of smokers’ spatial experience and context requires more ecological models. Virtual reality (VR) creates a state of immersion close to reality allowing controlled assessments of behavioral responses. To date, no studies investigated brain activation associated to smoking cue-reactivity in VR using electroencephalography (EEG).

**Aims:**

To investigate whether a VR cue-reactivity paradigm (a) may increase smoking craving, (b) is feasible with EEG recording, and (c) induces craving levels associated to EEG desynchronization.

**Methods:**

Smokers (*N* = 20) and non-smokers (*N* = 20) were exposed to neutral and smoking-related VR scenarios, without and with smoking conditioned stimuli, respectively. EEG was recorded from occipital and parietal leads throughout the sessions to assess alpha band desynchronization. Smoking and food craving and presence visual analogue scales (VAS) were assessed during the session.

**Results:**

To be smoker, but not non-smoker, significantly influenced smoking craving VAS induced by smoking cue VR but not by neutral VR. No significant food craving changes was observed during the VR sessions. The new finding was that EEG alpha band power in posterior leads was significantly increased by the smoking context scenario only in smokers, and that the degree of smoking (i.e., heavy vs. light) was significantly associated to this neurophysiological measure.

**Conclusions:**

This study demonstrated, for the first time, the feasibility of EEG recording in a VR setting, suggesting that EEG desynchronization may be a neurophysiological marker of smoking cue-reactivity.

## Introduction

Cue-reactivity is the vast array of responses that addicted people exhibit when exposed to conditioned and contextual stimuli previously associated to substance use (Chiamulera et al. 2017). Response to drug-related cue reactivity may be physiological (Drummond et al. 2000), psychological (Niaura et al. 1988) and behavioral (Rohsenow et al. 1991). Smoking cue-reactivity has been widely described by anecdotal, ecological, clinical, and experimental reports, and strongly related to risk of relapse to drug-seeking, i.e., cigarette smoking. For example, a smoking home suppresses the efficacy of pharmacotherapy for smoking cessation when compared to a smoking-free domestic environment suggesting that both discrete stimuli (such as objects) and living space could trigger relapses (Gilpin et al. 2006).

Imaging studies in humans showed the activation of brain areas involved in motivational, emotional, and cognitive processes when subjects are exposed to a drug-related cue (Yalachkov et al. 2012; McClernon et al. 2016). Smoking cue reactivity has been investigated by electroencephalography (EEG) studies, which showed greater alpha desynchronization to the presentation of conditioned stimuli compared to neutral ones in smokers (Cui et al. 2013). Frontal EEG activities have been reported to differ between smokers and non-smokers (Knott et al. 2008a; Rass et al. 2016). Taken together, these studies not only showed different EEG activation in smokers vs non-smokers, but also suggested that EEG could be a sensitive neurophysiological marker of cue reactivity.

The difficulty to recreate the complexity of smokers’ spatial experience and the context in a laboratory setting needs more ecological models. Virtual reality (VR), which creates a state of immersion closer to reality, allowing the controlled measure of neuropsychological and behavioral responses (Pericot-Valverde et al. 2015), may create an ecological and realistic experimental setting in human studies. For this reason, VR has been extensively used in drug and tobacco addiction, e.g., to explore smoking withdrawal, craving and cue reactivity (Pericot-Valverde et al. 2015). Although these VR reports confirmed findings previously demonstrated in traditional laboratory studies (i.e., cues presented as pictures or videos), better characterization of the cue reactivity phenomena triggered by VR simulation is needed. For instance, one psychological measure of experience in VR is the sense of presence (henceforth, presence), which was proposed to be related to allocation of attentional resources (Witmer and Singer 1998; see also the debate in Slater 1999) and correlated to EEG changes in sensorimotor integration areas (Vecchiato et al. 2015). The exploration of EEG activity in a cue reactivity VR experimental protocol may be a technically demanding issue. Indeed, the application of the VR helmet and the EEG cap on the head of the subjects and the eye, head, neck, and body movements associated with VR space exploration may potentially lead to a number of artifacts. To date, no studies investigated craving-related cortical activation for smoking in a VR ecological and controlled context using EEG.

The present study was aimed to investigate (a) whether a cue-reactivity paradigm may induce an increase in smoking craving in a controlled VR condition, (b) the feasibility of EEG recording in this experimental setting, and (c) if smoking craving level is associated to a specific pattern of EEG desynchronization. To these aims, smokers and non-smokers were exposed to a control (i.e., no smoking-related cues) VR scenario and then to smoking-related VR scenarios, with and without smoking conditioned stimuli. EEG was recorded from occipital and parietal leads throughout the VR scenarios to assess whether alpha band desynchronization was associated to smoking craving. Food craving and presence were also assessed during the session.

## Materials and methods

### Study design

**Fig. 1:**
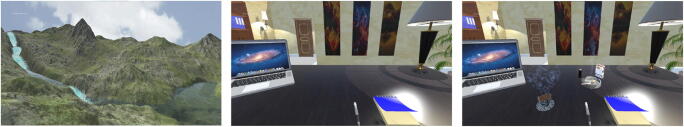
Screenshots of the virtual reality (VR) scenarios used in the study. Participants were exposed to a 3-min VR scenario (neutral, a mountain landscape; left panel), a 3-min VR office (control, non-smoking cues context; middle panel), and a 3-min VR office and smoking cues (smoking cue context containing conditioned stimuli; right panel)

Non-randomized study in two cohorts of smokers and non-smokers exposed to control and then smoking-related cues VR scenario on a fixed-block order procedure. These scenarios were chosen to represent a prototypic context, i.e., a room with the minimal number of office (control scenario), and office and smoking elements (smoking cues: ashtray, smoke, cigarette, pack of cigarette, cup of coffee; Fig. 1). The primary endpoint was the assessment of cue reactivity as craving level for smoking and food by using a visual analogue scale (VAS). Sense of presence was also assessed by using a VAS scale. Brain activation was measured by recording EEG during the presentation of VR scenarios.

### Subjects

Forty volunteers were recruited through online and paper advertisements, placed in the main gathering areas of the School of Medicine, Verona University, Italy. The procedures and potential risks associated to the experimental study were clearly and fully explained to the subjects. Furthermore, they were invited to read carefully and afterwards to personally fill out and sign an informed consent document, prior to the experimental sessions. The informed consent was in a language understandable to the subjects and clearly presented by one experimenter, who responded to all the questions raised by the subjects. The statement could be withdrawn in every moment and for every reason. The study was approved by the local academic ethical committee for research in healthy volunteers (*Comitato di Approvazione per la Ricerca sull*’*Uomo*, CARU, protocol code 22/2018), and followed the principles of the Declaration of Helsinki. The subjects’ data were collected and stored in a limited-access computer system, located in the Human Laboratory, Department of Diagnostic and Public Health, University of Verona, Italy. Each participant was given an identification code, formed by a number, subject’s initials, and date of birth to ensure the anonymity.

Inclusion criteria were as follows: (a) male or female, (b) age 18–65 years, and (c) should smoke at least 10 cigarettes a day, for at least the previous year (only for smoker group). Exclusion criteria were as follows: (d) personal or familiar history of seizures, (e) personal clinical history of cardiovascular or chronic disease, (f) current pregnancy, (g) cardiac pacemaker or any other metallic device or implant in the head-neck district, except piercings or braces, and (h) active therapy with psychotropic drugs, or use of psychoactive substances, which could interfere with the VAS or EEG measures. Inclusion and exclusion criteria were verified during an interview led by one of the experimenters prior to the study.

### Procedures

Subjects were welcomed in the Human Laboratory, Department of Diagnostic and Public Health, University of Verona, Italy. Smokers were requested not to smoke at least 1 h before the session. Smoking status was assessed by measuring the carbon monoxide (CO) expired concentration. Participants were invited to take place on the workstation chair, were instructed about the study procedures and risks, and signed the informed consent, and the experimenters confirmed the absence of exclusion criteria. All participants were instructed how to fill out the demographic and smoking questionnaires (smoker group only: smoking status-history assessment, Fagerström Test for Nicotine Dependence).

A standard 32 channels elastic cap of the appropriate size for the head of the patient was mounted.

**Fig. 2:**
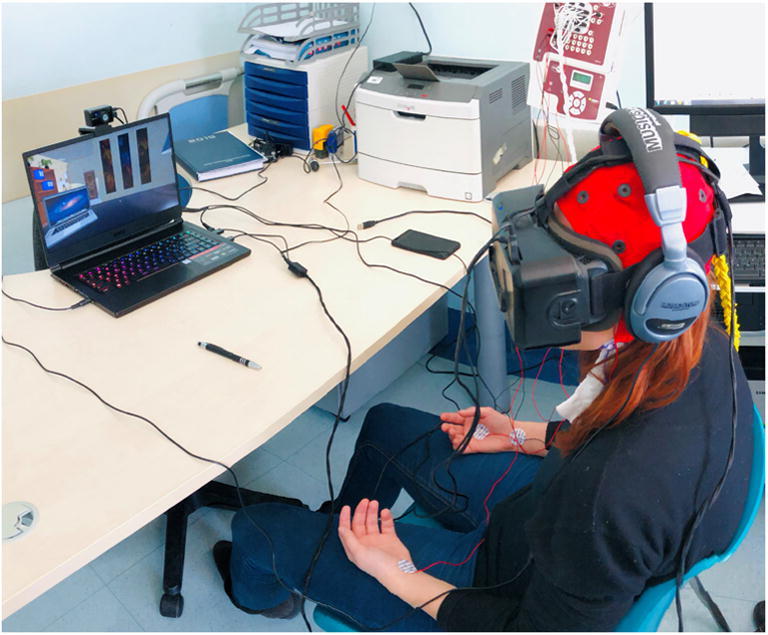
Experimental setup. Participants wore a standard EEG elastic cap, the virtual reality head-mounted display, and the headphones

**Fig. 3:**
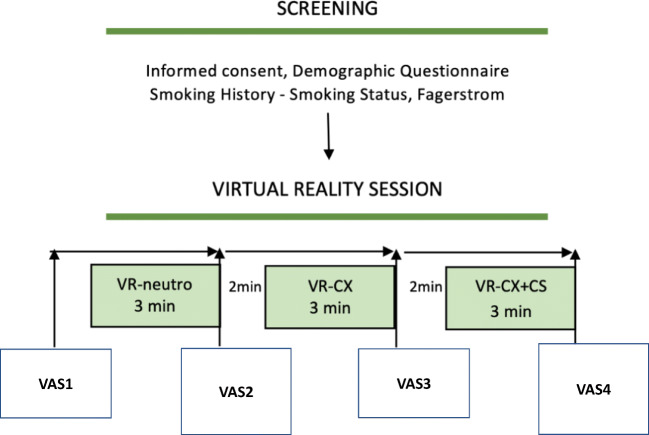
Schematic diagram of the study design. Each experimental session lasted about 45 min, including informed consent, screening procedure, baseline questionnaires, and virtual reality (VR) session. Visual analogue scale (VAS) for smoking craving, food craving, and sense of presence was filled out after the VR sessions. CX, context; CS, conditioned stimuli, i.e., smoking cues; VR-neutro, VR neutral scenario; VR-CX + CS, VR smoking context scenario

The experimenters gave instructions on how the head-mounted display worked and how to move in the VR environment. The experimenters helped the participant to wear the head-mounted display and headphones (Fig. 2). Participants were exposed to a 3-min VR scenario (neutral; a mountain landscape) followed after a 2-min pause without VR display by the 3-min VR office (control; non-smoking cues context) and then, after another 2-min pause, by the 3-min VR office and smoking cues (smoking context) containing conditioned stimuli (a smoking cigarette in an ashtray, a pack of cigarette, a coffee; Figs. 1, 3). The protocol was a fixed-block order procedure as recommended for avoiding craving carry-over effect (Sayette et al. 2010). Subjects were required not to do any specific task in the VR scenarios; they were left free to explore the surroundings with their head movement. At the end of each scenario, subjects removed the Oculus Rift VR display and filled out the smoking and food craving VAS questionnaire, which was also filled before the VR sessions. The presence questionnaire was filled after the neutral and at the end of the experimental procedure after the smoking context scenario. The study was a single session, lasting about 45 min.

### Virtual reality tools

Oculus Rift is a VR head-mounted display, which comprises a pair of eye goggles, positioned over the participant’s face, developed by Oculus VR a division of Facebook Inc. (Menlo Park, California, USA). The Rift is designed to provide a stereoscopic vision with a full 6 degrees of freedom environment. Thanks to its rotational and positional tracking system, Oculus Rift allows the subject to explore the scene by moving the head and body. Participants also wore a pair of headphones to simulate environmental sounds. VR scenarios were created using the development platform Unity.

### Questionnaires

The Smoking Status-History Assessment Questionnaire consists of five questions about the current status and the smoking history. The Fagerström Test for Nicotine Dependence is a widely used and extensively validated questionnaire to test the degree of nicotine dependence via tobacco smoking (Heatherton et al. 1991). The Fagerström score was evaluated according to Heatherton et al. (1991), where low dependence is represented by score 0–2, low-to-moderate dependence by score 3–4, moderate dependence by score 5–7, and high dependence by score ≥ 8.

The 0–10 VAS for smoking and food craving was adapted from Traylor et al. (2011). VAS for sense of presence was adapted from Vecchiato et al. (2015). All the VASs were scored before VR sessions (VAS1) and after the three scenarios (VAS2-4).

### EC50 Smokerlyzer

The expired CO concentration, a non-invasive method of assessing smoking status, was measured by using the EC50 Smokerlyzer (Bedfont Instruments; Kent, UK). Breath CO levels was expressed in parts per million (ppm) based on the conversion of CO to carbon dioxide (CO_2_) over a catalytically active electrode (Middleton et al. 2000).

### EEG

EEG activity was recorded during VR sessions with a digital Galileo NT (EB Neuro, Florence, Italy) system, using active Ag/AgCl electrodes at 32 scalp sites according to the International 10/10 system (ACNS 2006), i.e., 32 standard channels mounted on an elastic cap and two mastoid locations, M1 and M2, which were used for off-line re-referencing. All signals were digitized with a sample rate of 256 Hz. Offline, the EEG signals were referenced to the mathematically linked mastoids, and EEG and EOG were phase-shift-free filtered using a 0.1–30 Hz band-pass filter and a notch (50 Hz) filter. Impedance was kept below 10 KOhm.

### Sample size

The sample size for this study was calculated using G*Power software version 3.1.5.1 (Faul et al. 2009) with *α* = 0.05 and power (1-*β*) = 0.8. The estimated sample size estimated was 20 participants for each group.

### Statistical analysis

Statistical analyses were performed using the IBM SPSS version 20.0 and the PRISM 6 software (GraphPad Software Inc., San Diego, CA, USA).

Two-way rm-ANOVA with between-group factor status (two levels: non-smokers, smokers) and within-group factor scenario (four levels: VAS1, VAS2, VAS3, VAS4) was applied to smoking and food craving VAS, followed by post hoc with Sidak’s test.

Two-way rm-ANOVA with between-group factor status (two levels: non-smokers, smokers) and within-group factor scenario (three levels: VAS2, VAS3, VAS4) was applied to presence VAS measure, followed by post hoc with Sidak’s or Dunnett’s tests.

EEG data were processed using the EEGLAB (Delorme and Makeig 2004) version 14 software. Artifacts correction was performed using baseline correction and independent components analysis technique (ICA; Makeig et al. 1996). Each EEG segment underwent Fast Fourier transform (FFT) using a Hanning window of 10%. For each VR scenario, the FFTs were averaged and alpha (7.75–13.75 Hz) band power from O1, O2, P3, P4, and Pz electrodes was log_10_-transformed. Three-way rm-ANOVA with between-group factor status (two levels: non-smokers, smokers) and within-group factor scenario (three levels: scenario1, scenario2, scenario3) and electrode (five levels: O1, O2, P3, P4, and Pz) was applied to alpha band power, and post hoc *t* test with Sidak’s test.

Subjects in the smoker group were further divided into light and heavy smokers according to the number of daily smoked cigarettes (< 10 and ≥ 10/day, respectively), and rm-ANOVA was further performed with the between-group factor status2 (three levels: non-smokers, light smokers, heavy smokers).

For all the analyses, the statistical significance was set at *p* < 0.05 (two-tailed).

## Results

### Demographics

The demographic data are reported in Table 1.

**Table 1:** Demographic characteristics of the non-smoker and smoker groups

	Non-smokers		Smokers		Total	
Characteristic	*N*	%	*N*	%	*N*	%
Sex						
Male	10	50.0	13	65.0	23	57.5
Female	10	50.0	7	35.0	17	42.5
Age						
≤ 25	19	95.0	19	95.0	38	95.0
26–30	1	5.0	1	5.0	2	5.0
Highest degree awarded						
High school diploma	20	100.0	18	90.0	38	95.0
Bachelor’s degree	-	-	2	10.0	2	5.0
Years of smoking						
≤ 10	-	-	20	100.0		
Number of cigarettes/day						
< 10	-	-	13	65.0		
10–15	-	-	6	30.0		
> 15	-	-	1	5.0		
Fagerström score						
Low dependence	-	-	18	90.0		
Low-to-moderate dependence	-	-	1	5.0		
Moderate dependence	-	-	1	5.0		
High dependence	-	-	-	-		

Age, mean = 22.78, SD = 1.98. No subject of the non-smoker group reported to be an ex-smoker. The Fagerström score was evaluated according to Heatherton et al. (1991), where low dependence is represented by score 0–2, low-to-moderate dependence by score 3–4, moderate dependence by score 5–7, and high dependence by score ≥ 8

### Smoking craving

**Fig. 4:**
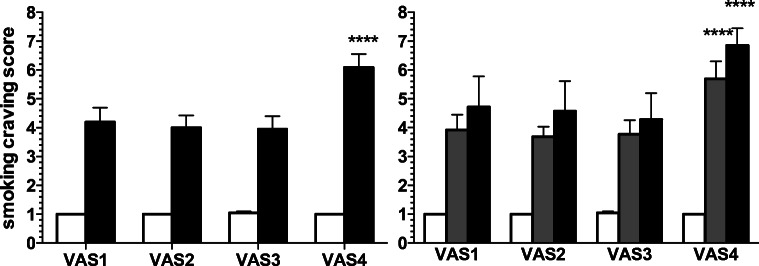
Smoking craving. *Left panel*. Smoking craving score in the non-smokers (open columns; *N* = 20) and the smokers (solid columns; *N* = 20) groups at the different timepoints (VAS1 to VAS4). *Right panel*. Smoking craving score in the non-smokers (open columns; *N* = 20), light smokers (gray columns; *N* = 13), and heavy smokers (solid columns; *N* = 7) groups at VAS1 to VAS4. Data are expressed as mean + SEM. ****Significant within-group post hoc (*p* < 0.0001 vs. VAS1, VAS2, and VAS3; Sidak’s test). Between-groups significant post hoc comparisons are reported in the main text

Two-way rm-ANOVA showed significant effect of status (*F*[1.38] = 75.36; *p* < 0.0001), scenario (*F*[3.114] = 18.95; *p* < 0.0001) and significant status × scenario interaction (*F*[3.114] = 19.69; *p <* 0.0001). Post hoc within-group comparisons showed that the smokers, but not the non-smoker group, reported significantly higher craving score at the last timepoint (VAS4) compared to the other timepoints (VAS1 to VAS3; *p* < 0.0001 for all comparisons; Sidak’s test; Fig. 4). Post hoc between-group comparisons showed significant higher craving score in the smokers vs. non-smoker groups for all timepoints (VAS1 to VAS4; *p* < 0.0001 for all the comparisons; Sidak’s test).

Two-way rm-ANOVA showed a significant effect of status2 (*F*[2.37] = 39.61; *p* < 0.0001), scenario (*F*[3.111] = 29.91; *p* < 0.0001), and significant status2 × scenario interaction (*F*[6.111] = 10.03; *p* < 0.0001). Post hoc within-group comparisons showed that the light smokers and the heavy smokers, but not the non-smokers, had significantly higher VAS4 craving score compared to VAS1-3 (*p* < 0.0001 for all comparisons; Sidak’s test; Fig. 4). Post hoc between-group comparisons showed significant higher craving score in the light smokers vs. non-smokers and heavy smokers vs. non-smoker groups for all timepoints (VAS1 to VAS4; *p* < 0.0001 for all the comparisons; Tukey’s test; Fig. 4).

### Food craving

**Fig. 5:**
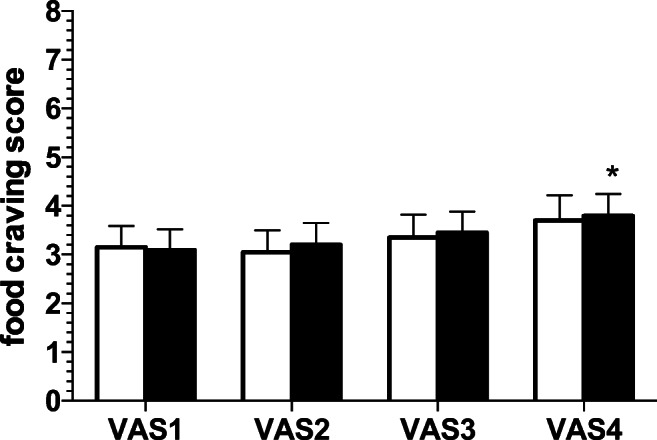
Food craving. Food craving score in the non-smokers (open columns; *N* = 20) and the smokers (solid columns; *N* = 20) groups at the different timepoints (VAS1 to VAS4). Data are expressed as mean + SEM. *Significant within-group post hoc (*p* < 0.05 vs. VAS1; Dunnett’s test)

Two-way rm-ANOVA showed a significant effect of scenario (*F*[3.114] = 5.83; *p* < 0.001) but not of status (*F*[1.38] = 0.02; NS) and no interaction (*F*[3.114] = 0.12; NS). Post hoc within-group comparisons showed that the smokers, but not the non-smokers, had significantly higher VAS4 craving score compared to baseline VAS1 (*p* < 0.05; Dunnett’s test; Fig. 5). Post hoc between-group comparisons yielded no significant differences at all timepoints (NS; Sidak’s test).

### Presence

**Fig. 6:**
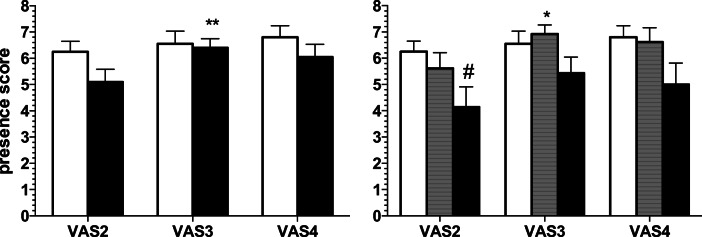
Sense of presence. *Left panel*. Presence scores in the non-smokers (open columns; *N* = 20) and the smokers (solid columns; *N* = 20) groups at different timepoints (VAS2 to VAS4). *Right panel*. Presence scores in non-smokers (open columns; *N* = 20), light smokers (gray columns; *N* = 13), and heavy smokers (solid columns; *N* = 7) groups at VAS2 to VAS4. Data are expressed as mean + SEM. *p < 0.05 and **p < 0.01 significant within-group post hoc vs. VAS2; Sidak’s test. #Significant between-groups post hoc (*p* < 0.05 vs. VAS2 in non-smokers; Dunnett’s test)

Two-way rm-ANOVA showed significant effect of scenario (*F*[2.76] = 4.53; *p* < 0.05), but not of status (*F*[1.38] = 1.70; NS) and no significant interaction (*F*[2.76] = 1.43; NS). Post hoc within-group comparisons showed that the smokers, but not the non-smokers, had a slight but significant presence score increase at VAS3 vs. VAS2 timepoint (*p* < 0.01; Sidak’s s test; Fig. 6). Post hoc between-group comparisons yielded no significant differences at all timepoints (NS; Sidak’s test).

Two-way rm-ANOVA showed a significant main effect of scenario (*F*[2.74] = 4.88; *p* < 0.01) but not of status2 (*F*[2.37] = 2.02; NS) and no interaction (*F*[2.74] = 0.70; NS). Post hoc within-group comparisons showed a significant increase of presence in the light smokers at VAS3 vs. VAS2 timepoint (*p* < 0.05; Sidak’s s test; Fig. 6). Post hoc between-group comparisons showed a significantly lower presence score at VAS2 when comparing heavy smokers to non-smokers (*p* < 0.05; Dunnett’s test).

### Alpha band power

**Fig. 7:**
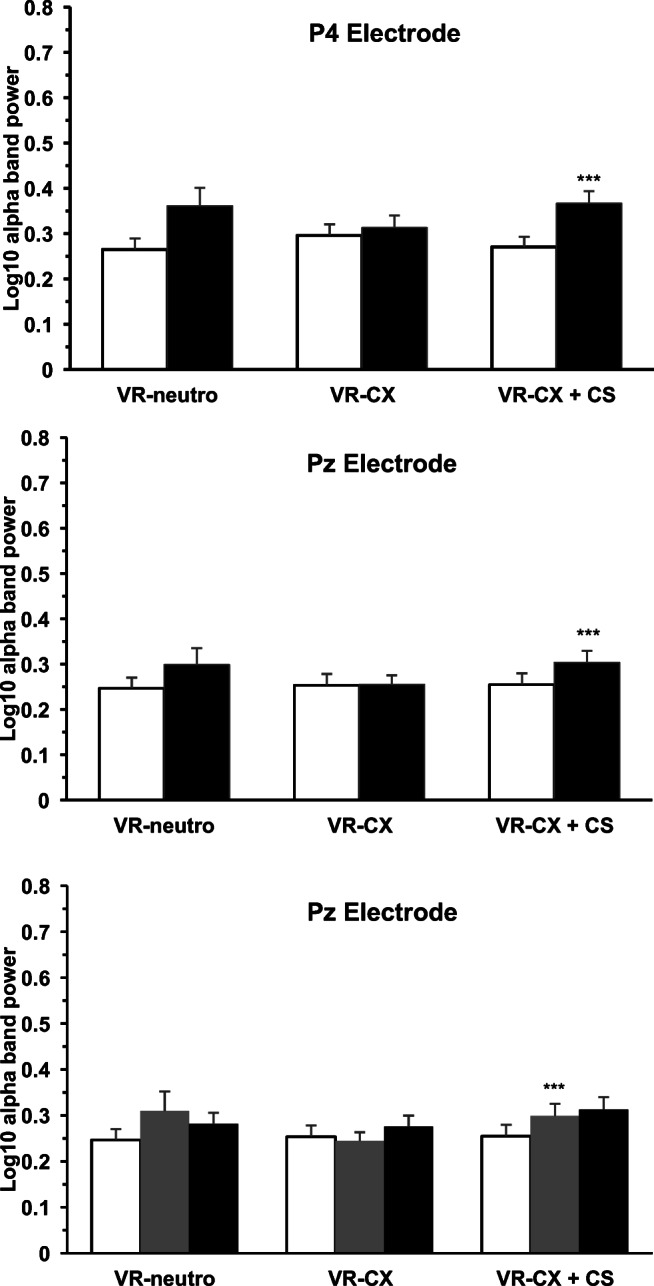
EEG Alpha band power. *Upper/central panel*. Log_10_ alpha band power in the non-smokers (open columns; *N* = 20) and smokers (solid columns; *N* = 20) groups during 3-min VR mountain landscape scenario (VR-neutro), 3-min VR non-smoking cues context office (VR-CX), and 3-min VR smoking cue context + conditioned stimuli (VR-CX + CS) measured at P4 and Pz electrodes. *Lower panel*. Log_10_ alpha band power in non-smokers (open columns; *N* = 20), light smokers (gray columns; *N* = 13), and heavy smokers (solid columns; *N* = 7) measured at Pz electrode. Data are expressed as mean + SEM. ***Significant within-group post hoc (*p* < 0.01 vs. VR-CX; Sidak’s test)

Three-way ANOVA showed a significant main effect of electrode (*F*[4.304] = 16.9; *p* < 0.001), and significant electrode × status (*F*[4.304] = 3.6; *p* = 0.008) and scenario × status (*F*[2.304] = 4.5; *p* = 0.014) interaction on the log_10_ alpha band power. Post hoc within-group comparisons showed significantly higher alpha band power during scenario 3 in comparison to scenario 2 in P4 (*p* = 0.002; Sidak’s test) and Pz (*p* < 0.001; Sidak’s test) in the smokers, but not the non-smokers (Fig. 7).

Three-way ANOVA showed a significant main effect of electrode (*F*[4.296] = 16.4; *p* < 0.001), and significant electrode × status (*F*[8.296] = 2.4; *p* = 0.019) and scenario × status (*F*[4.296] = 3.7; *p* = 0.008) interaction on the log_10_ alpha band power. Post hoc within-group comparisons showed significantly higher alpha band power during scenario 3 in comparison to scenario 2 in PZ (*p* = 0.003; Sidak’s test) in light smokers only (Fig. 7).

## Discussion

As expected, to be a smoker, but not a non-smoker, significantly influenced smoking craving induced by exposure to the smoking cue scenario (VAS4), but not by neutral and control scenarios. The absence of increasing smoking craving during the session when no smoking cues were presented excluded that higher craving scores at the end of the session was due to passage of time or to pre-session cigarette craving carry-over effect. Our data are in agreement with VR literature on smoking cue reactivity (Lee et al. 2003; Baumann 2004; Baumann and Sayette 2006; García-Rodríguez et al. 2011) and smoking context (Garcia-Rodriguez et al. 2012; Paris et al. 2011). To be heavy or light smoker was not associated to differences in smoking craving, suggesting that the number of daily smoked cigarettes is not correlated to smoking cue reactivity under our VR procedure. Furthermore, our results confirm the validity of the model, since no significant food craving changes was observed during the sessions. The small but significant increase in the smoker group after the last scenario suggests however a gradual increase in appetitive urges.

The increase of presence during the experimental session in the smoker group could be due to a pre-existing lower presence trait at baseline. This hypothesis is in line with data from a previous study, where we found a similar lower presence score in smokers compared to non-smokers independent from type of VR scenario (unpublished data). This finding is however opposite in direction to studies in alcohol (Bordnick et al. 2008) and cocaine addicts (Rosenthal et al. 2007) that showed an increased presence in addicts, whereas Gamito et al. (2014) did not observe any significant interaction between smoking status and presence.

A new and interesting finding of the present study was that EEG alpha band power in posterior leads was significantly increased by the smoking context scenario only in smokers, and that the level of smoking (i.e., heavy, light) had a significant effect on this neurophysiological measure. Some previous studies explored EEG in smokers and during smoke cue-reactivity experimental protocols. Daily smokers showed reduced resting delta and alpha EEG power (Rass et al. 2016). Frontal theta band power was found to be decreased during smoking cue reactivity, similarly to a condition of withdrawal, in males with no change in frontal alpha band power (Knott et al. 2008b). Frontal alpha EEG was reported to be asymmetric (i.e., left frontal hypoactivation) with cigarette-cue exposure, particularly in female smokers, with both decreases and increases in brain state during cigarette-cue exposure, influenced by depressed mood (Knott et al. 2008a). A significant increase in beta power, but no changes in delta and theta activity, between smoke-related cue and neutral stimuli, together with reduction of theta power in reward craving and increase in left posterior alpha in withdrawal craving, was observed in smokers (Littel et al. 2009). Finally, emotional and cigarette-related stimuli were reported to induce less alpha power than neutral stimuli in smokers (Cui et al. 2013). Comparison of our data to those previously published is difficult, because of the differences in study protocols, EEG leads and bands explored, and the presence/absence of smoking withdrawal across studies. A limitation of this study is that it was not possible to evaluate the possible correlation between the increased alpha band in posterior lead for smokers exposed to smoking context and the presence level. Increased parietal alpha power has been correlated to higher presence in VR (Pfurtscheller and Lopes da Silva 1999; Kober et al. 2012), suggesting the involvement of parietal areas in the sense of spatial presence. This view is in keeping with evidence that egocentric spatial processing in parietal areas is expression of body-centered visual control of location (Baumgartner et al. 2006, 2008). Another limitation stems from the limited range of Fagerström scores in our sample of smokers, in that the large majority of them had low dependence impeding a comparison between participants with different dependence levels.

Indeed, the present study demonstrated, for the first time, that EEG recording is feasible in a VR setting, suggesting that EEG may represent a neurophysiological marker of smoking cue-reactivity that may be used in a highly ecological experimental setting.
